# Effects of Graphene Nanoplates on the Mechanical Behavior and Strengthening Mechanism of 7075Al Alloy

**DOI:** 10.3390/ma13245808

**Published:** 2020-12-19

**Authors:** Jinfeng Leng, Yunfan Dong, Binghui Ren, Ran Wang, Xinying Teng

**Affiliations:** School of Materials Science and Engineering, University of Jinan, No. 336, Jinan 250022, China; dyf777777@163.com (Y.D.); renbinghui694@163.com (B.R.); ran_2wang@163.com (R.W.); mse_tengxy@ujn.edu.cn (X.T.)

**Keywords:** graphene nanoplates, 7075Al, stirring casting, strengthening mechanisms

## Abstract

7075Al alloy is the preferred material for lightweight automotive applications, but the existing problem is that it is difficult to combine high strength and high toughness. This paper presents our research aimed at obtaining high strength and high toughness materials by adding a nano-phase to realize microstructure refinement. Graphene nanoplates (GNP)/7075Al composites and 7075Al alloy were prepared by a stirring casting method in the present study. In comparison to 7075Al, the tensile strength of GNP/7075Al composites was increased from 572 MPa to 632 MPa while maintaining good uniform elongation of 8% to 10%. The increased strength behavior of GNP/7075Al composites while maintaining the plasticity is discussed in terms of grain refinement and dislocation evolution by analyzing the composite microstructure and quantitatively analyzing the contributions of grain boundary strengthening, solid solution strengthening, precipitation strengthening and dislocation strengthening. GNP’s strengthening of GNP/7075Al composites is mainly attributed to the refinement of grain size and the increase of dislocation density.

## 1. Introduction

7xxx series aluminum alloy is the preferred material for lightweight automobile applications, but the traditional 7xxx series alloy has encountered bottleneck problems in having both strength and plasticity [1,2]. The refinement of the alloy microstructure by adding nanoparticles achieves the goal of high strength and toughness [3], which provides a solution to the above problems. Graphene is a two-dimensional material with special mechanical and physical properties [4]. It is considered an ideal reinforcement material for metal matrix composites due to its low density, high specific surface area, ultra-high Young’s modulus and strength [5,6,7]. In addition, two-dimensional graphene is more easily dispersed in the aluminum matrix than one-dimensional carbon nanotubes. In recent years, with the upgrading of graphene preparation technology and the advancement of large-scale industrialization, it has become possible for high-quality and low-cost graphene to be applied in the field of composite materials [8].

The preparation methods of graphene-reinforced aluminum matrix composites (AMCs) have been explored, but at present the main preparation methods are spark plasma sintering, hot pressing, etc. Ankita et al. [9] prepared 1 wt.% GNS/Al through a spark plasma sintering process, whereby the yield strength and tensile strength increased by 84.5% and 54.8% respectively, but the elongation decreased from 3.5% to 0.6%. Zhang et al. [10] prepared 0.5% and 1.0 wt.% GNP/Al5083 by hot pressing and heating extrusion processes. Compared with Al5083, the yield strength increased by 68 MPa and 113 MPa, respectively, but the elongation decreased from 6.6% to 4.6% and 3.0%. Wang et al. [11] prepared 0.3 wt.% GNS/Al by a vacuum hot pressing process. Compared with the unenhanced Al, the tensile strength increased from 154 MPa to 249 MPa, but the elongation decreased from 27% to 13%. Zhao et al. [12] prepared 0.2 vol.% rGO/Al by vacuum hot pressing and rolling processes. The tensile strength increased from 221 MPa to 277 MPa and the elongation decreased from 12.1% to 6.5% compared with Al. Obviously, high strength and high plasticity cannot be realized simultaneously by using a nano-reinforcer in the above preparation processes. Stir casting technology belongs to liquid phase preparation technology and materials with high strength and high plasticity can be obtained under good interfacial layer control. At the same time, stir casting processes represent the earliest commercial production process of metal matrix composite materials, which can be used for continuous and semi-continuous large-scale production with traditional equipment [13,14,15].

Al-Zn-Mg-Cu alloys are common precipitation-strengthened alloys. In this class of precipitation-strengthened alloys, very small and uniformly dispersed precipitates are formed in the Al matrix after heat treatment, which blocks dislocation motion and thus enhances the strength ofthe alloys [16,17,18]. However, there have been few reports on the role of GNP in al-Zn-Mg-Cu alloys and the potential effects of graphene addition on other strengthening mechanisms, which include solid-solution strengthening, grain-boundary strengthening, precipitation strengthening and dislocation strengthening. Therefore, the goal of present study was to quantify the enhancement mechanism by directly comparing the 7075Al alloy and the graphene/7075 aluminum matrix composite.

In this paper, graphene nano-platelets (GNP) reinforced AMCs were prepared by a stirring casting process. The relationship between the properties and microstructure of GNP-reinforced AMCs was explored, and the contribution of GNP to various strengthening mechanisms was quantitatively calculated.

## 2. Experimental

The primary materials were Al ingots (99.7%), Mg ingots, Zn ingots, Al–50Cu master alloys, A1-4Ti master alloys, Al-5Cr master alloys and less than 5 layers thick GNP.

**Figure 1:**
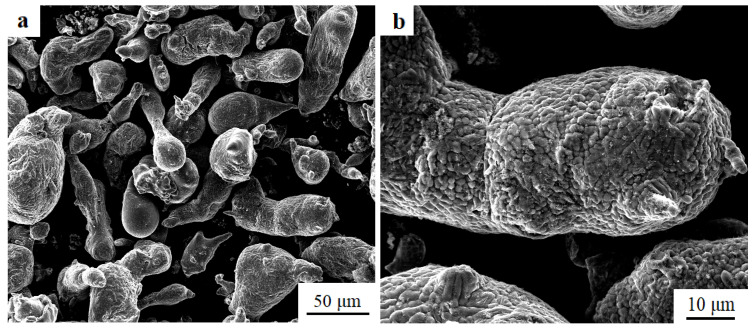
SEM images of (**a**,**b**) mixed powder of GNP and Al powder.

The GNP and Al powders with a particle diameter of 200 mesh (74 μm) were blended in a planetary ball mill machine under an argon atmosphere at 150 rpm for 10 h with a ball to powder ratio of 10:1 (Figure 1). The mixed powders were blown into the melt by an inert gas and then the melt was stirred evenly and keep still for 20 min, after which the melt was cast. For comparison, an unreinforced 7075Al alloy was also fabricated using the same method. The nominal compositions of two materials are listed in Table 1.

A series of heat treatment processes were then carried out. Homogenization annealing of ingot casting in 465 °C for 16 h was carried out. Then, the billets were heated at 470 °C for 1 h and extruded into bars of an extrusion ratio of 10:1 with an extrusion rate of 0.3 mm/s. In order to obtain the ultimate performance, the extrusion bars were solution treated at 470 °C for 1 h, water nd athen aged at 120 °C for 24 h (T6).

The static tensile property was measured by a WDG-1060 electronic universal testing machine (Jinan Kesheng Test Equipment Co. LTD, Jinan, China) with a tensile rate was 2 mm/min at room temperature. The microstructures were observed using a 4 × MC optical microscope after being polished and etched with Keller solution of HF (1 mL), HCl (1.5 mL), HNO_3_ (2.5 mL) and H_2_O (95 mL). The grain size of T6 GNP/7075Al and T6 7075Al was estimated at least 150 grains in metallographic images by using the Image-J software. The precipitate size was also estimated from the HRTEM images by the Image-J software. The metallographic structure of the test materials was observed by a QUANTA 250 scanning electron microscope (FEI, Zaragoza, Spain) at 20 KV. Phase composition was observed on a D8 Advance X-ray diffractometer (Bruker, Billerica, MA, USA) using Cu-Kα radiation. The sample was imaged in a JEM-2010TEM (JEOL, Tokyo, Japan) operated at 200 kV.

## 3. Results and Discussion

### 3.1. Microstructures of the Composites

**Figure 2:**
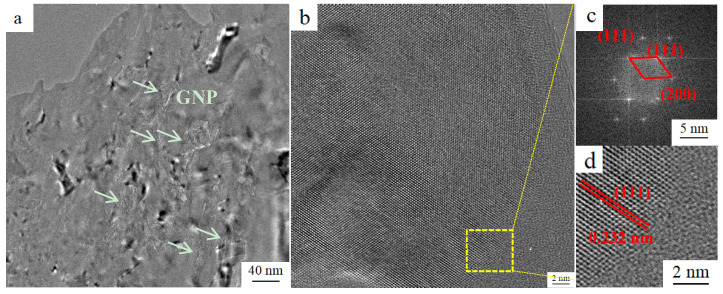
(**a**) TEM and (**b**) HRTEM images of GNP/7075Al; (**c**) FFT and (**d**) IFFT of yellow area in (**b**).

A typical TEM image of T6 GNP/7075Al composite is shown in Figure 2a. It can be seen that the GNP was tightly embedded into the Al matrix. At the interfaces between GNP and Al matrix, a very strong bonding was observed. The good wettability between GNP and Al matrix leads to close interface bonding without distinct crevices. Figure 2b is HRTEM of the interface between Al and GNP. The moire fringes is observed around the interface. The formation of moire fringes could be attributed to the formation of high density of dislocations. Figure 2c,d are FFT (Fourier transform) and IFFT (Inverse Fourier transform) images recorded from the marked box (yellow box in Figure 2b) shows (−111), (200) and (111) diffraction spots of Al matrix and lattice fringes with the measured inter-planar spacing of 0.61 nm, which corresponds to the plane of (111). A good interface between Al matrix and GNP can be seen in Figure 2d.

**Figure 3:**
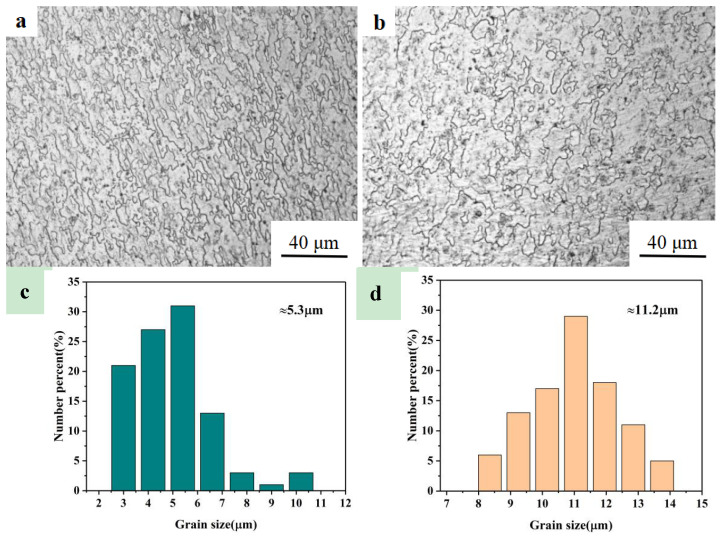
Metallography of (**a**) T6 GNP/7075Al and (**b**) T6 7075Al; and corresponding grain sizes of (**c**) GNP/7075Al and (**d**) 7075Al.

In order to analyze the effect of GNP addition on mechanical properties, the microstructure of GNP/7075Al composite and 7075Al alloy was analyzed. Figure 3 shows metallography and a histogram of the grain size distribution of (a) T6 GNP/7075Al composite, and (b) T6 7075Al alloy, respectively. It shows that the original grains were plastically elongated perpendicular to the compression direction. The average grain sizes of GNP/7075Al composites and 7075Al alloy are 5.3 μm and 11.2 μm, respectively. The grain size obtained by statistics is basically the same as that of some materials with the same heat treatment process. For example, the average grain sizes of 7085Al alloy, SiC/7085Al composite and SiC/7085Al-1.0 Mg composite prepared by hot pressing and T6 heat treatment were 3.4 μm, 3.1 μm and 3.8 μm, respectively [19].

According to our previous study, the molten aluminum nucleates on the GNP, resulting in a grain size of GNP/7075Al composite that is smaller than that of 7075Al alloy during the aluminum melt crystallization process [20]. In addition, the presence of GNP limits the growth of Al grains in the plastic deformation during the hot extrusion process of GNP/7075Al composite materials, resulting in the decrease of grain size of the composite materials. Grain refinement improves the strength and plasticity of the material when the grain size is micron. Therefore, the strength and plasticity of GNP/7075Al composites prepared by stirring casting heating extrusion process can be improved compared with that of GNP/7075Al alloy.

**Figure 4:**
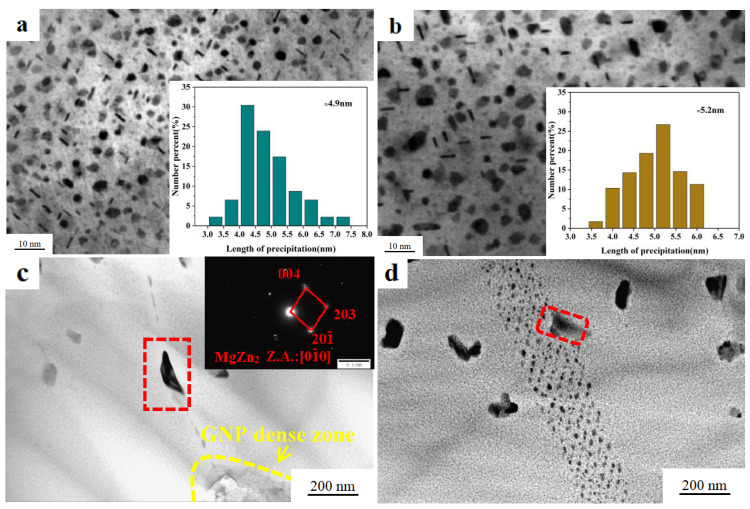
Bright-field TEM images and precipitate size statistic plots of (**a**) GNP/7075Al composite and (**b**) 7075Al alloy; Bright-field TEM images of (**c**) GNP/7075Al composite, (**d**) 7075Al alloy.

The addition of GNP results in grain refinement and also affects the precipitate phase inside the grain. Figure 4 depicts the TEM images of GNP/7075Al composite and 7075Al alloy. The morphology, size and number density of GP zones, η′ and η-phase precipitates were different between the T6 GNP/7075Al composite and 7075Al alloy. A large number of solute atoms are separated from the aluminum lattice at the early aging stage and aggregated to form GP zones. Part of GP zones are transformed into η′ phase instead of η phase with the aging process. The main strengthening precipitates are extremely fine GP zones and η′ phase. The subsequent strengthening mechanism analysis requires the precipitation phase’s mean length *l* and mean edge-to-edge interprecipitate spacing *λ_p_*, so we estimated it from 10 representative TEM images. Both GNP/7075Al composite and 7075Al alloy have a large number of nanometer GP zones with an average diameter of 3–4 nm, and the bar-shaped η′ precipitation phase. Figure 4a,b show that the precipitates in the GNP/7075Al composite and 7075Al alloy had a mean length of 4.9 nm and 5.2 nm, respectively. The addition of GNP slightly refines the precipitated phase because the lattice defect introduced by addition of GNP is the preferred nucleation site for heterogeneous nucleation. Most of η phase (red circle) is mainly formed in grain boundary and subboundary, while the strengthening phase of GNP/7075Al composites and 7075Al—η′ phase—is mainly formed in grain (Figure 4c).

**Figure 5:**
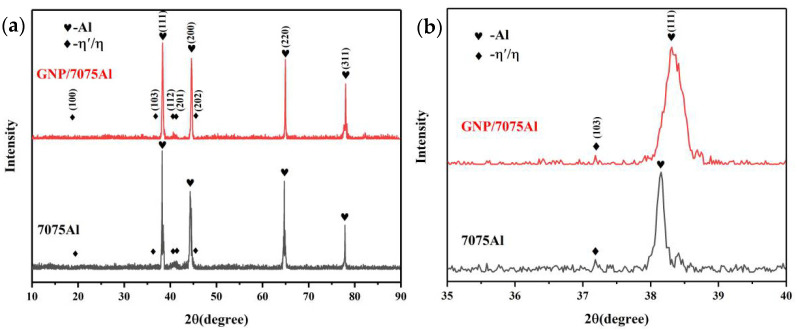
(**a**) X-ray diffraction patterns for GNP/7075Al composite and 7075Al alloy and (**b**) High magnification in area (35°–40°).

In order to further analyze the phase composition of GNP/7075Al composites and 7075Al alloy and calculate the lattice distortion and dislocation density, X-ray diffraction analysis was carried out on it. Figure 5 shows the XRD patterns of T6 GNP/7075Al composites and 7075Al alloy.

It can be seen from the XRD patterns that there are obvious Al peak and weak η′-phase peak, because GNP/7075Al composite and 7075Al alloy basically contain a large number of GP zones and η′-phase with small volume fraction and dispersion distribution that corresponds to the study of Kaka [21]. Substitute Bragg Angle (*θ*) corresponding to the crystal surface of the strongest peak Al (111) into Bragg formula and calculation formula of crystal surface spacing. Comparing with Al’s standard PDF card, the lattice distortion of GNP/7075Al composites and 7075Al alloy calculated are 0.2%, 0.5%, respectively.

Based on XRD, the Williamson-Hall method can be used to calculate the dislocation density (*ρ*) in metal materials. According to the Williamson-Hall method, the broadening of the XRD diffraction peak is caused by the material’s microstrain which caused by dislocation, solute atoms and grain size changes [22,23]:

(1) $$B=B_{D}+B_{e}$$

where *B* is the full width at half-maximum (FWHM) of the peaks, *B_D_* is FWHM caused by grain size and *B*_e_ is FWHM caused by microstrain. The diffraction peak width caused by average effective microstrain (*e*) can be expressed as:

(2) $$B_{e}=2e\tan\theta$$

where *e* is average effective microstrain and *θ* is Bragg Angle. The diffraction peak width caused by grain size (*D*) can be expressed as:

(3) $$B_{D}=\frac{K\lambda }{D\cos\theta }$$

*K* = 0.9 is a shape factor, *λ* = 0.15406 nm is the wavelength of X-rap source and *D* is grain size. Substitute Equations (2) and (3) into Equation (1):

(4) $$Bcos\theta =\frac{K\lambda }{D}+2e\sin\theta$$

Kril et al. [24] have shown that the change of grain size has a significant effect on the XRD peak width of the material when the grain size of the material is less than 100 nm. At present work, grain size of GNP/7075Al composite and 7075Al alloy which are prepared by stirring casting and hot extrusion in micron level. Therefore 1/D is going to be relatively small and negligible. Equation (4) can be expressed as:

(5) Bcosθ≈2esinθ

If the strain is caused only by dislocation, the relationship of dislocation density (*ρ*) to the average effective microstrain (e) can be expressed as [19]:

(6) $$\rho =\frac{14{.4e}^{2}}{b^{2}}$$

where *b* is the Burgundy vector. The dislocation densities for GNP/7075Al composite and 7075Al alloy are calculated to be 1.1 × 10^15^ m^−2^ and 4.1 × 10^14^ m^−2^, respectively. The dislocation density calculated in present work is similar to that of some studies on plastic deformation of aluminum alloy. For example, Liu et al. [25] obtained the dislocation density of ECAP 6013 aluminum alloy at different temperatures as 1.2–1.7 × 10^14^ m^−2^ through XRD diffraction. Schiller et al. [26] measured the dislocation density of ECAP 7000 series Al alloys at 200 °C by XRD to be 3.4 × 10^14^ m^−2^. The addition of GNP results in GNP/7075Al composites having a higher dislocation density than 7075Al alloys. The thermal expansion coefficients of GNP and Al matrix are −3.5 × 10^−^^6^/K and 23.2 × 10^−^^6^/K respectively. Because graphene and aluminum matrix cannot deform together, high residual stress will be generated at the interface when the processing temperature drops to room temperature. In the subsequent aging process, the release of residual stress is manifested as the generation of a large number of dislocations.

**Figure 6:**
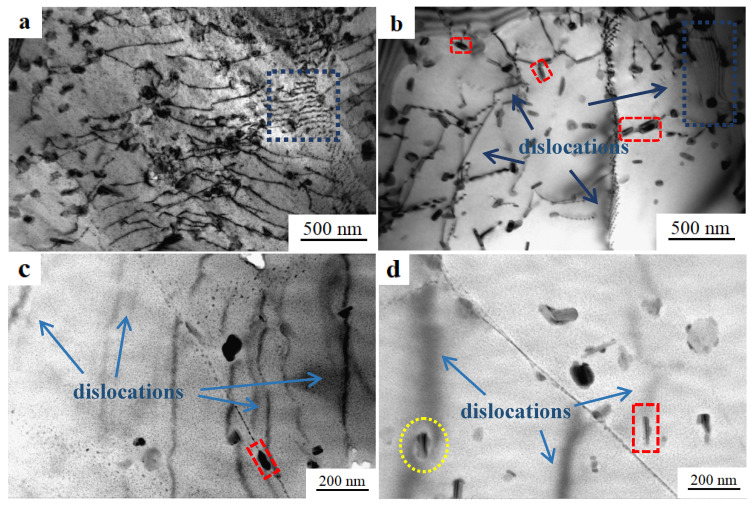
Bright-field TEM images of (**a**,**c**) GNP/7075Al composite, (**b**,**d**) 7075Al alloy.

Figure 6 shows bright-field TEM images of GNP/7075Al composites and 7075Al alloy. A large number of dislocations are arranged along the direction of compression, and dislocation plug set is seldom produced (Figure 6a,c). It can be seen that the dislocation density of GNP/7075Al composites is higher than the dislocation density of 7075Al alloy and the dislocation is punctured by a black second phase (red box). Consequently, Orowan dislocation bypassing has been determined to be the operative strengthening mechanism for all of the second-phase particles. Figure 6d shows that a small number of dislocations and the precipitate phase (yellow circle) which is circumvented by dislocation and rod-shaped precipitation phase with sizes of 200 nm inside the grain, which is generally considered to be Al_2_CuMg or Al_18_Cr_2_Mg_3_ phase, which produced in the casting process and does not disappear in the subsequent heat treatment [27].

### 3.2. Mechanical Properties

**Figure 7:**
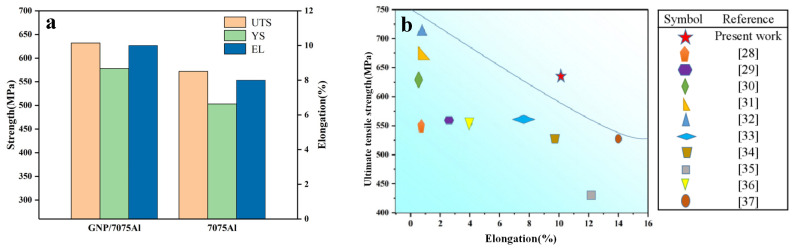
(**a**) Tensile properties of GNP/7075Al composite and 7075Al alloy and (**b**) a comparison of the ultimate tensile strength and elongation of GNP/7075Al composite fabricated in the present study and previous reports.

The mechanical properties of T6 GNP/7075Al composites and 7075Al alloy determined by tensile tests are shown in Figure 7a. The yield strength and tensile strength of T6 7075Al alloy are 503 MPa and 572 MPa, respectively. Compared with 7075Al alloy, the yield strength and tensile strength of T6 GNP/7075Al composites increased by 15% and 10%, respectively, reaching 578 MPa and 632 MPa. The elongation of GNP/7075Al composite and 7075Al alloy are 10% and 8%, respectively, indicating that when the amount of GNP added is 0.2wt.%, it can not only improve the strength, but also has no adverse effect on the plasticity. Synchronous enhancements in strength and ductility were obtained in GNP/7075Al composites (T6). A comparison of the ultimate tensile strength and elongation of GNP/7075Al composite fabricated in the present study and previous reports in Figure 7b [28,29,30,31,32,33,34,35,36,37]. It can be concluded that the GNP/7075Al composite exhibit high strength and good ductility compared with previously developed aluminum matrix composites.

**Figure 8:**
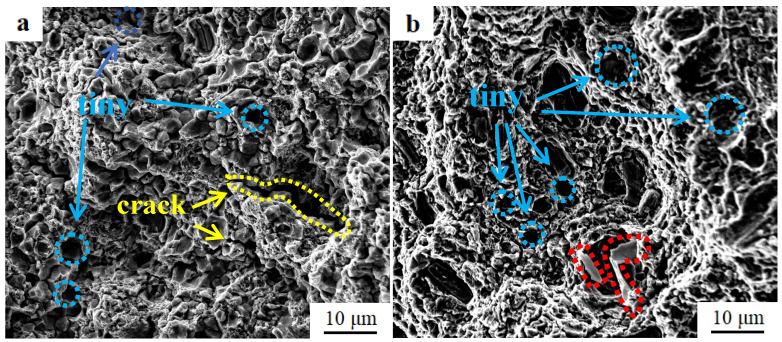
Fracture morphology of (**a**) GNP/7075Al composite and (**b**) 7075Al alloy.

The typical magnified fractography of GNP/7075Al composite and 7075Al alloy after T6 treatment is presented in Figure 8. A large number of tiny dimples and cracks extending into the grain (yellow circle) are presence in Figure 8a. Protruding precipitating phase from the dimple (red circle) and a large number of tiny dimples are presence in Figure 8b. The tensile fracture of GNP/7075Al composite and 7075Al alloy is mainly shear fracture with a small part along the crystal fracture.

### 3.3. Strengthening Mechanism

In order to analyze the contribution of GNP to the mechanical properties of 7075 aluminum alloy, the yield strength of GNP/7075Al composite can be divided into several parts [38,39,40,41]: solid-solution strengthening mechanism, grain-boundary strengthening mechanism, precipitate/ dispersoid strengthening mechanism, dislocation strengthening mechanism and load transfer mechanism. The physical meaning and values of the symbols in the equations used in this study are shown in Table 2 [38,42,43].

Solute atoms are dissolved into the crystal lattice, which causes the crystal lattice distortion and increases the anti-slip property, thus enhancing the strength and hardness. The functions of solute elements are mainly elastic interactions, chemical interactions and electronic interactions. Solution strengthening (*Δσ_ss_*) is represented by the Fleischer equation [44]:

(7) $$\Delta \sigma_{\mathrm{ss}}=MGb\varepsilon^{\frac{3}{2}}{}_{ss}\sqrt{c}$$

*M* is orientation factor of polycrystalline matrix; *G* is the shear modulus and *ε* is lattice strain. It can be seen from equation 7 that *Δσ_ss_* depends on the shear modulus difference and lattice strain between solute and matrix, which is mainly related to solute concentration and the size difference between solute and matrix atoms. GNP/7075Al composite and 7075Al alloy primarily contains Zn, Mg and Cu solute atoms, whose contents are shown in Table 1. The contribution of atoms Zn, Mg and Cu to yield strength are 2.9, 18.6 and 13.8 MPa/wt.% [21]. It can be seen that the contribution of Zn, Mg and Cu solute added by GNP/7075Al composite and 7075Al alloy to yield strength are 16.24 MPa, 48.36 MPa and 20.01 MPa, respectively, and the total contribution to yield strength is 84.8 MPa. Considering that solute atoms (Zn, Mg, Cu) do not completely dissolve in the lattice of Al and some of them precipitate out during aging process. The contribution to yield strength provided above is an upper limit, and its true contribution to yield strength is lower than 84.8 MPa. In the above study, it has been found that the addition of GNP reduced the lattice distortion from 0.5% to 0.2%, that is, more solute atoms would precipitate out at the grain boundary rather than inside the lattice of Al, thus resulting in the weakening of the solid solution strengthening effect.

The finer the grain, the more grain boundaries, the stronger the resistance to dislocation motion. The grain-boundary strengthening mechanism is usually described by the Hall-Petch equation [21]:

(8) $$\sigma_{y}=\sigma_{0}+\frac{k_{y}}{\sqrt{d}}$$

where *σ_0_* is the friction stress, *d* is the average grain diameter and *k_y_* is the Hall-Petch slope. Maung et al. [45] prepared aluminum matrix composites by isometric trap extrusion method, and found that the Hall-Petch behavior changed to the inverse Hall-Petch behavior when the grain size was less than 110 nm. However, the grain size of the material prepared by stirring casting process is far more than 110 nm, so the strengthening mechanism is still Hall-Petch behavior. Wert et al. [38] studied the effect of grain size on yield strength of 7xxx series aluminum alloys and revealed that the Hall-Petch coefficient *k_y_* of T6 7075Al was 0.12 MPa/m^1/2^. Assuming that the *σ_0_* values of GNP/7075Al composite and 7075Al alloy are the same and the increased yield strength *Δσ_y_* is proportional to *d^−1/2^*, the mean grain sizes of T6 GNP/7075Al composite and 7075Al alloy above are substituted into Equation (8). The increase in yield strength of GNP/7075Al composite and 7075Al alloy due to grain-boundary strengthening is calculated to be 52 MPa and 36 MPa.

The solid solution atoms are precipitated from the Al lattice to form a precipitation phase in the aging process, which hinders the movement of the dislocation line, so as to improve the strength. There are two main strengthening mechanisms of precipitation strengthening: (i) the dislocation bypasses the precipitation phase and consumes the dislocation energy, thus hindering the dislocation movement; (ii) the dislocation cuts through the precipitation phase to consume the dislocation energy, thus hindering the dislocation movement. The main strengthening phase of T6 GNP/7075Al composite and 7075Al alloy is η′ phase, so the precipitation strengthening mechanism is Orowan dislocation bypassing. *Δσ_orowan_* could be calculated by the following equation [40,42]:

(9) $$\Delta \sigma_{orowan}=M\frac{0.4Gb}{\pi \sqrt{1-\upsilon }}\frac{\ln(2\bar{r}/b)}{\lambda_{p}}$$

where *M*, *G*, *b*, *υ λ_p_* are listed in Table 2; $\bar{r}=\sqrt{2/3r}$, where r is the mean radius of the precipitates. For simplified calculation, the precipitates phase is assumed to be spherical, *r = l/π*, where *l* is 4.9 nm and 5.2 nm, corresponding to GNP/7075Al composite and 7075Al alloy, respectively. According to the mean length *l* and mean edge-to-edge interprecipitate spacing *λ_p_* of GNP/7075Al composite and 7075Al alloy, the contributions to yield strength can be calculated as 388 MPa and 361 MPa, respectively.

The mutual hindrance between the dislocations increase the strength of the material. The contribution of the dislocation strengthening (*Δσ_q_*) of GNP/7075Al composite and 7075Al alloy is usually calculated by the Taylor equation [46]:

(10) $$\Delta \sigma_{q}=M\alpha Gb\rho^{\frac{1}{2}}$$

where *M*, *α*, *G*, *b*, *ρ* are listed in Table 2. The contributions to yield strength cause by dislocation can be calculated as 156 MPa and 95 MPa for GNP/7075Al composite and 7075Al alloy, respectively.

Since no cracks are observed in Figure 2b, it is shown that the compact and carbide free GNP/Al interface is strong enough to give full play to the strengthening effect of GNP. The good combination of GNP/Al interface allows the transfer of payload from Al matrix to GNP. Based on the shear-lag model, the load-transfer contribution to the yield strength improvement (*Δσ_LT_*) could be estimated as [12,47]:

(11) $$\Delta \sigma_{LT}=\alpha^{\prime }V_{g}\sigma_{g}-V_{g}\sigma_{m}$$

where *V_g_* is the volume fraction of GNP, *σ_g_* is the yield strength of GNP and *σ_m_* is yield strength of matrix. The expression of the factor $\alpha^{\prime }=\frac{\tau_{m}S}{2\sigma_{g}}$ [47]. In this case, load transfer mechanism (*Δσ_LT_*) in the GNP/7075Al composites was estimated to be 11.9 MPa.

The strength increments stemming from the four mechanisms are summarized in Table 3. It can be seen that the strength enhanced by precipitate strengthening mechanism is the strongest in the five strengthening mechanisms, followed by the dislocation strengthening. The addition of GNP enhanced grain boundary strengthening, precipitated phase strengthening and dislocation strengthening, and reduced solid solution strengthening.

## 4. Conclusions

The effects of GNP on the microstructure, mechanical properties and strengthening mechanisms of GNP/7075Al composites prepared by stirring casting were investigated. The results showed that in comparison to 7075Al, the grain size of GNP/7075Al composites decreased from 11.2 μm to 5.3 μm. The HRTEM observations indicate that there is a good interface between GNP and the Al matrix, which is conducive to load transfer. Furthermore, GNP induces a large number of dislocations at the interface.

The GNP/7075Al composite exhibit an improved tensile strength (from 572 MPa to 632 MPa), yield strength (from 503 MPa to 578 MPa) and good ductility (elongation from 8% to 10%) simultaneously. According to theoretical calculations, the strengthening mechanisms of GNP/7074Al composites are mainly grain-boundary strengthening, precipitate strengthening, dislocation strengthening and load transfer strengthening, which are 52, 388, 156 and 11.9 MPa, respectively. In comparison to 7075Al, the increased effects of strengthening mechanism in GNP/7075Al composites caused by GNP are mainly shown in grain-boundary strengthening, dislocation strengthening and load transfer strengthening.

## Figures and Tables

**Table 1:** Nominal composition of GNP/7075Al and 7075Al (in wt.%).

Composites	Zn	Mg	Cu	Cr	Ti	Fe	Si	GNP	Al
GNP/7075Al	5.6	2.5	1.6	0.2	0.2	<0.1	<0.1	0.2	Bal
7075Al	5.6	2.5	1.6	0.2	0.2	<0.1	<0.1	0	Bal

**Table 2:** Physical meaning and values of different symbols used in the strengthening mechanism calculation.

Symbol	Meaning	Values
*M*	Mean orientation factor	3.06 for the fcc (Face Center Cubic)
*b*	Magnitude of the Burgers vector	0.286 nm for fcc Al
*G*	Shear modulus	26.9 GPa for Al 7075
*α*	Constant	0.2 for fcc metals
λ_p_	mean edge-to-edge interprecipitate spacing (GNP/7075Al and 7075Al)	20.5 nm; 22.7 nm
*k_y_*	Hall-Petch coefficient	0.12 MPa/m^1/2^
*υ*	Poisson ratio	0.33 for Al 7075
*d*	mean grain sizes (GNP/7075Al and 7075Al)	5.3 μm; 11.2 μm
*l*	Mean length of η′ (GNP/7075Al and 7075Al)	4.9 nm; 5.2 nm
*ρ*	dislocation density (GNP/7075Al and 7075Al)	1.1 × 1015 m^−2^; 4.1 × 1014 m^−2^

**Table 3:** Estimated strength increment for different strengthening mechanisms.

	σ_(Solid Solution)_	σ_(Grain Boundary)_	σ_(Precipitation)_	σ_(Dislocation)_	σ_(Load Transfer)_
GNP/7075Al	<<84 MPa	52 MPa	388 MPa	156 MPa	11.9 MPa
7075Al	<84 MPa	36 MPa	361 MPa	95 MPa	-
